# Design and comparison of a hybrid to a traditional in-person point-of-care ultrasound course

**DOI:** 10.1186/s13089-022-00261-x

**Published:** 2022-03-12

**Authors:** Michael Janjigian, Anne Dembitzer, Caroline Srisarajivakul-Klein, Aron Mednick, Khemraj Hardower, Deborah Cooke, Sondra Zabar, Harald Sauthoff

**Affiliations:** 1 Department of Medicine, New York University Grossman School of Medicine, NYC Health & Hospitals/Bellevue, Bellevue Hospital Center, New York, USA; 2 Department of Medicine, New York University Grossman School of Medicine, NY Harbor Healthcare System, New York, USA; 3 Department of Medicine, New York University Grossman School of Medicine, NYU Langone Health, New York, USA

**Keywords:** Point-of-care ultrasound, Medical education, Program assessment

## Abstract

**Background:**

Traditional introductory point-of-care ultrasound (POCUS) courses are resource intensive, typically requiring 2–3 days at a remote site, consisting of lectures and hands-on components. Social distancing requirements resulting from the COVID-19 pandemic led us to create a novel hybrid course curriculum consisting of virtual and in-person components.

**Methods:**

Faculty, chief residents, fellows and advanced practice providers (APPs) in the Department of Medicine were invited to participate in the hybrid curriculum. The course structure included 4 modules of recorded lectures, quizzes, online image interpretation sessions, online case discussions, and hands-on sessions at the bedside of course participant’s patients. The components of the course were delivered over approximately 8 months. Those participants who completed a minimum of 3 modules over the year were invited for final assessments. Results from the hybrid curriculum cohort were compared to the year-end data from a prior traditional in-person cohort.

**Results:**

Participant knowledge scores were not different between traditional (*n* = 19) and hybrid (*n* = 24) groups (81% and 84%, respectively, *P* = 0.9). There was no change in POCUS skills as measured by the hands-on test from both groups at end-of-course (76% and 76%, respectively, *P* = 0.93). Confidence ratings were similar across groups from 2.73 traditional to 3.0 hybrid (out of possible 4, *P* = 0.46). Participants rated the course highly, with an average overall rating of 4.6 out 5.

**Conclusions:**

A hybrid virtual and in-person POCUS course was highly rated and as successful as a traditional course in improving learner knowledge, hands-on skill and confidence at 8 months after course initiation. These results support expanding virtual elements of POCUS educational curricula.

**Supplementary Information:**

The online version contains supplementary material available at 10.1186/s13089-022-00261-x.

## Background

Point-of-care ultrasound (POCUS) reduces procedural complications, improves diagnostic accuracy and increases provider and patient satisfaction, leading to the expansion of POCUS beyond emergency departments and intensive care units [1–6]. In addition, the availability of less expensive ultrasound devices and the increasing accessibility of training programs have facilitated the growth of POCUS to medical students, residents in internal medicine and family practice, as well as to Advanced Practice Providers (APPs).

The Society of Hospital Medicine (SHM) advocates for attendance at a local or national POCUS training program, followed by a longitudinal study phase with hands-on instruction [7]. Traditional introductory POCUS courses are resource intensive, typically requiring 3 days at a remote site, consisting of lectures and hands-on components. These barriers are particularly limiting in low- and middle-income countries (LIMC), where there may exist a paucity of on-site POCUS experts, equipment, and high-speed internet access [8]. We recently reported the design and evaluation of the Integrated Sonographic Course at NYU (New York University) (I-ScaN) faculty POCUS training program showing that a 2-day introductory course followed by a year-long longitudinal curriculum led to sustained gains in hands-on skill and confidence with only a slight decay in knowledge [9]

The COVID pandemic disrupted nearly all aspects of medical education due to social distancing requirements. However, the benefits of POCUS in the care of COVID-19 and non-COVID patients compelled us to continue to train more clinicians by adapting our POCUS course to meet institutional requirements and minimize risk of COVID-19 transmission [10]. We describe the design, implementation and 1-year outcomes of our hybrid I-ScaN POCUS course as compared to our traditional course cohort.

## Methods

### Setting and participants

**Fig. 1:**
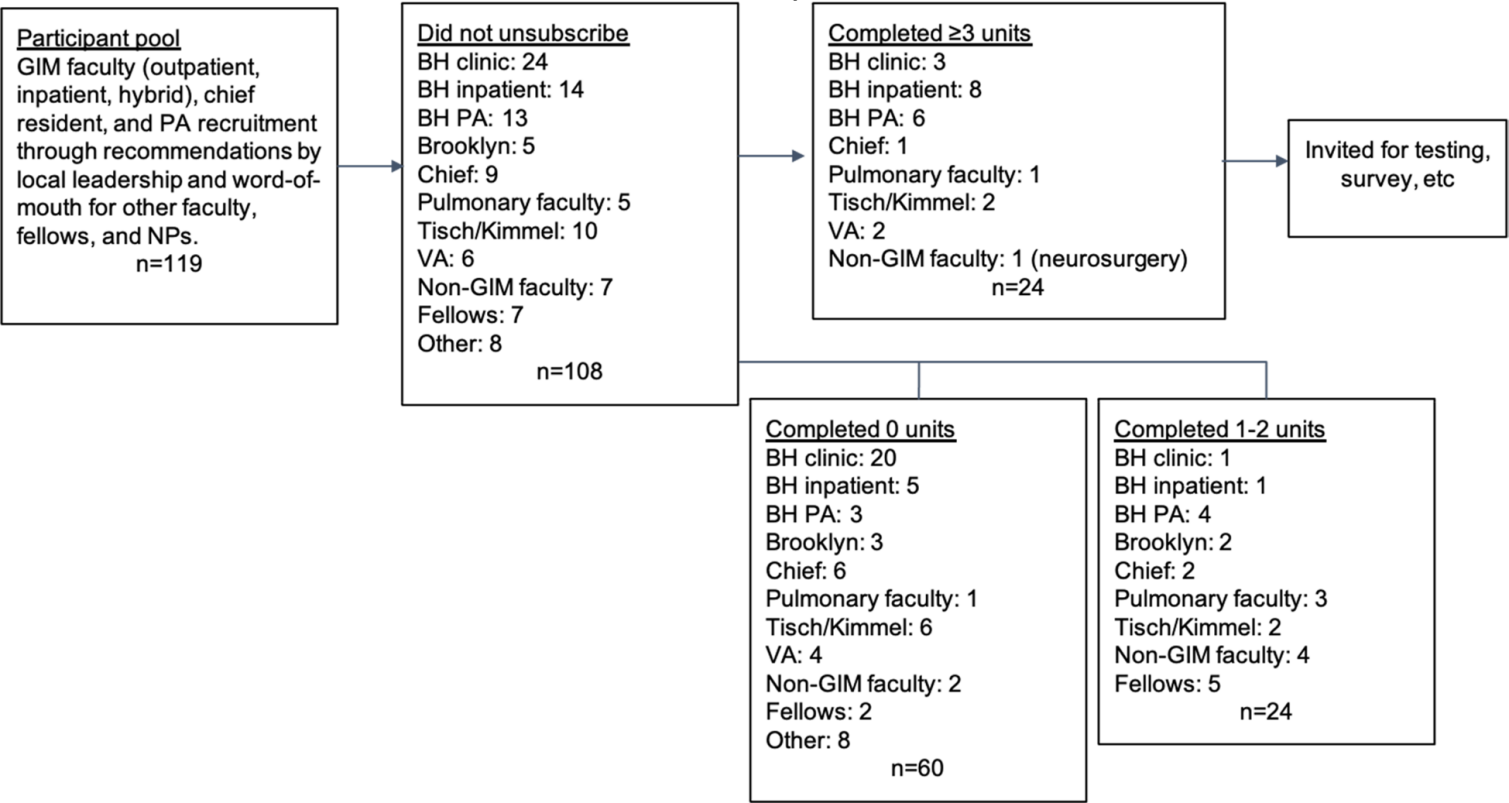
Participant progression through course. GIM: General Internal Medicine, BH: Bellevue Hospital

The NYU Grossman School of Medicine academic system spans four teaching hospitals, NYU Langone Health (Tisch/Kimmel and Brooklyn campuses), Health + Hospitals/Bellevue, and the New York Harbor Health Care System Margaret Cochran Corbin VA Campus. Potential participants were identified through recommendation by divisional leadership at each site, targeting outpatient and inpatient physicians, and inpatient APPs. Additional participants were identified through self-referral from faculty in other divisions, from faculty in other departments, from fellows in Medicine specialties, and from APPs not initially identified. This group of 119 candidates was emailed a course invitation with an option to unsubscribe if they had no intention of participating. Figure 1 shows the breakdown of participants offered entry to the course, those who did not unsubscribe but did not participate, those who completed 1–2 course elements, and those who completed ≥ 3 course elements.

### Program description

**Fig. 2:**
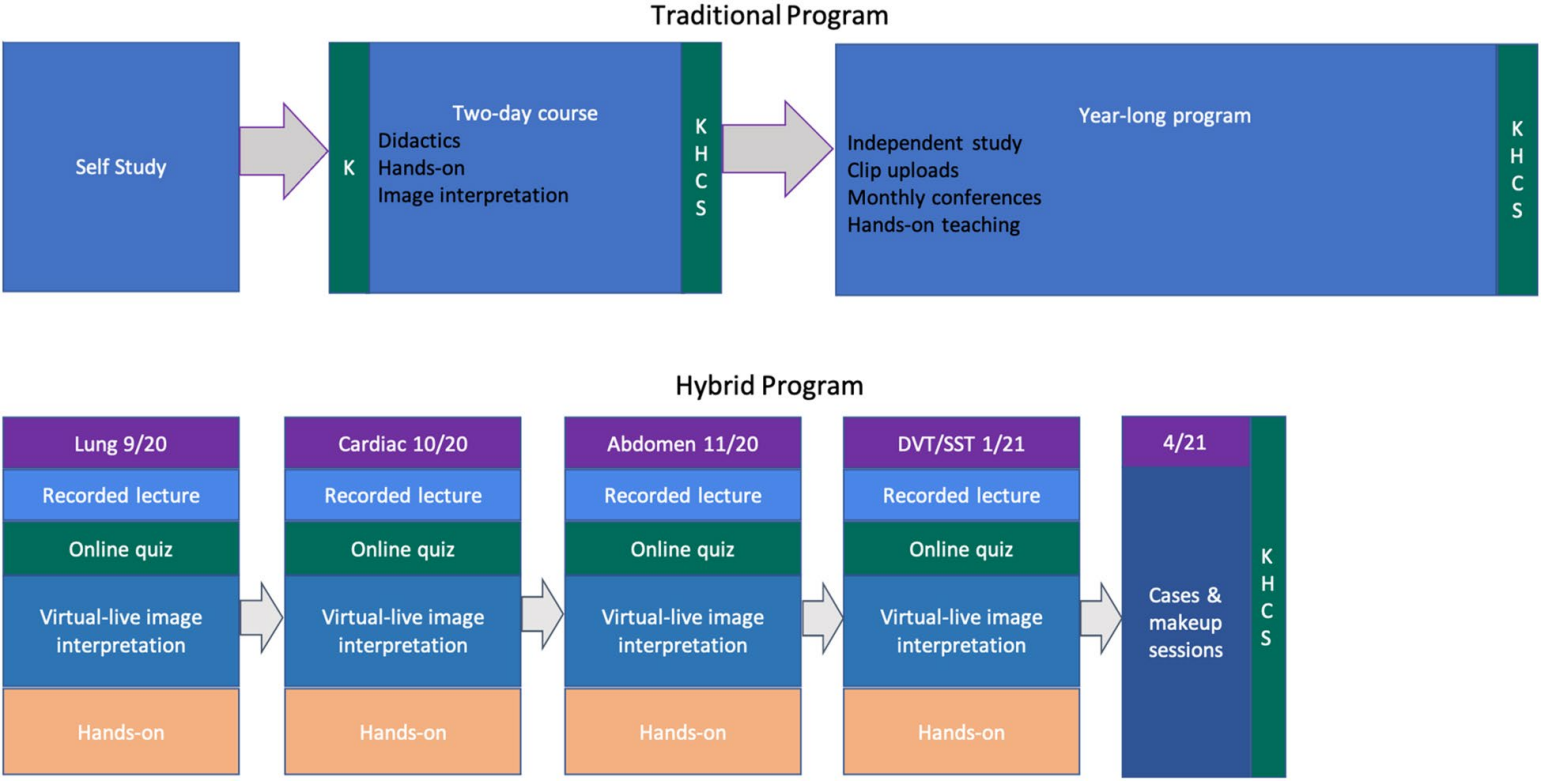
Timeline of course components and assessments for the Traditional and Hybrid programs. K: Knowledge test, H: Hands-on test, C: Confidence survey, S: Satisfaction survey, DVT: Deep Venous Thrombosis, SST: Skin and Soft Tissue

The traditional I-ScaN program has already been described in detail [9]. Participants studied included 19 hospitalists from the 4 NYU sites who completed the final assessments in 2019. The traditional course started with a 1-month self-study period, where participants were referred to relevant chapters from a POCUS textbook, selected articles, and online videos [11]. The 2-day in-person introductory course is modeled on the American College of Chest Physicians Critical Care Ultrasound course [12]. Systems covered in the course included cardiac (five standard views), lungs/pleura, abdomen (kidneys, bladder and aorta) and leg vasculature. Each system is taught with a didactic lecture reviewing theoretical concepts, an interactive image-based review of normal and abnormal findings, and hands-on training on a human model with a faculty to learner ratio of no more than 1:3. The longitudinal phase of the program began immediately after the 2-day course, consisting of monthly conferences, directly supervised hands-on scanning sessions, and self-directed practice with clip uploads with expert review (Fig. 2).

To adapt for COVID-19, a course structure was developed that included four modules based on the above organ systems, each consisting of a recorded lecture, an online quiz, an interactive online image interpretation session, and an in-person hands-on session, all followed by two 1-h case discussions held online (Fig. 2). The course began in September of 2020 with each module lasting approximately 1 month followed by case reviews, a period of make-up sessions, and ending with final assessments in April–May of 2021.

Lectures were recorded by the same instructors from the traditional course and uploaded to Brightspace (D2L), an online education platform. To add an interactive feature, each lecture was paired with a quiz containing an educational feedback section hosted on Brightspace to be completed after watching the video. Image interpretation sessions were hosted online by a course instructor, each held multiple times over a few weeks to accommodate participant schedules. Hands-on teaching sessions were conducted with appropriate infection control precautions with no more than three learners at the bedside of a patient typically under the care of one of the participants.

Participants were notified via email of each online session and asked (but not required) to watch the lecture and take the quiz in advance. Following the completion of the four modules, make-up online and hands-on sessions were held for those unable to attend earlier sessions. Only those completing the image interpretation session were invited for hands-on teaching for that module.

### Program evaluation

POCUS skills were evaluated using a hands-on test with human models and proctored by course faculty. All course participants and standardized patients were required to be fully vaccinated against COVID-19 and wear appropriate personal protective equipment (PPE). Knowledge of POCUS principles, image acquisition, and clinical integration was assessed using a 26-item online test. Participant confidence in image acquisition and clinical integration was assessed using a retrospective pre–post-survey [9]. All assessments were administered at course completion using the same tools and methodology as with the traditional cohort.

Participants completed a questionnaire regarding overall satisfaction with the program and individual elements. Two focus groups were conducted by one of the authors (AD) who is not a course instructor, discussing attitudes towards POCUS and reflections on the course.

The I-ScaN program qualified as a quality improvement project by the NYU Grossman School of Medicine’s Institutional Review Board criteria using a self-certification process to ensure the data were not collected for research purposes. The primary goal of the project was to assess and improve educational performance of the I-ScaN program.

Scores from knowledge were reported as percentages, confidence scores reported on a 1–4-point scale, and hands-on assessment reported as % well done. Differences between traditional and hybrid group variables were assessed using 2-sample Wilcoxon rank sum tests. Cronbach’s rating was consistent between groups and instruments.

## Results

The 24 participants completing at least 3 of the 4 modules were invited for final assessments approximately 8 months after beginning the course, with 83% (20/24) completing the knowledge test and confidence survey, and 79% (19/24) completing the hands-on test (see Table 1). Comparison of the final assessments between the hybrid (8 months after beginning the course) and traditional cohorts (12 months after beginning course) were performed. Participant knowledge results showed no significant difference between traditional and hybrid groups (81% and 84% correct, respectively, *P* = 0.9). There was no difference in POCUS skills as measured by the hands-on test from both groups at end-of-course (76% for both, *P* = 0.93). Confidence ratings were not statistically different from 2.73 traditional to 3.0 hybrid (out of possible 4, *P* = 0.46).

**Table 1:** Results of end-of-course assessments for the Traditional and Hybrid cohorts

	Traditional Median (IQR)	Hybrid Median (IQR)	P
Knowledge (% correct)	81 (24)	84 (15)	0.90
Confidence (1–4 Likert)	2.73 (0.78)	3 (0.47)	0.46
Hands-on (% well done)	76 (30)	76 (14)	0.93

*IQR* Interquartile range

Results of the hands-on test for the specific skills learned are reported in Table 2 and results of participant confidence regarding those skills are reported in Table 3.

**Table 2:** Comparison of hands-on test scores between Traditional and Hybrid cohorts

	Traditional (avg)	Hybrid (avg)
Parasternal long axis	2.5	2.7
Parasternal short axis	2.6	2.5
Apical 4 chamber	2.5	2.3
Subcostal long	2.2	2.1
Inferior vena cava	2.7	2.2
Lung A-line pattern	2.9	2.9
Right kidney	2.5	3.0
Left kidney	2.6	2.4
Bladder	2.7	2.8
Aorta short axis	2.1	2.4
Leg vasculature*	2.3	2.6

Test scored on a 3-point scale of (1) Poorly done (2) Partly done (3) Well done

*Average combined score for common femoral vein above, at, and below the greater saphenous vein, lateral perforator vein and popliteal vein

**Table 3:** Comparison of confidence scores in acquiring images between Traditional and Hybrid cohorts

	Traditional (avg)	Hybrid (avg)
Cardiac	2.9	3.2
Lung	3.3	3.2
Abdomen	3.1	2.9
Leg vasculature	2.4	2.9

4 point Likert scale of 1 not at all confident to 4 very confident

Participants rated the course highly, with an average overall rating of 4.6 out 5. In free text comments and in feedback provided during focus groups, participants reported liking having access to uploaded lectures that could be rewatched as often as needed and felt the online sessions were valuable. Participants would have liked to have more hands-on teaching.

We held focus groups to explore why some faculty chose not to attend the course, while others fully participated. The focus group of faculty who had not participated in any course elements but remained on the course mailing list included two outpatient faculty and two hospitalists. The hospitalists felt POCUS was directly relevant to their clinical care and teaching. The outpatient faculty did not feel POCUS was necessary for their clinical practice, but were interested to keep abreast of what their colleagues, residents and students were learning. Barriers to beginning the course were primarily related to lack of time for both outpatient faculty and hospitalists. All participants reported having too many responsibilities with clinical and non-clinical duties, compounded by the ongoing COVID pandemic. They also reported hesitancy to begin the course due to not fully understanding the commitments expected of them. The option of a 2-day course that could be blocked with advanced notice was appealing to some, though others felt they may not be allowed to block time or that it would not be feasible given their various responsibilities. The hospitalists reported that they would plan to take the hybrid course if offered again now that the pressures of COVID are receding and they have more control over their schedules.

The focus group of participants who completed all course elements included four hospitalists, a chief resident, an inpatient PA, and an outpatient attending. Most reported their motivation to take the course was that POCUS is becoming standard of care clinically, that students and residents are learning it and expect to be supervised and taught, and that POCUS improves diagnostic accuracy and time to diagnosis. The outpatient faculty acknowledged not planning on using POCUS in clinical practice, but wanted to learn the language of POCUS and to pursue a new skill. Participants uniformly praised having lectures available on Brightspace which they watched and rewatched during the course, and whenever a refresher was needed during clinical practice. The modular format supported learning through spacing and repetition, building layers of knowledge and skills that solidified over time. Online image review sessions could be done, while at work or at home, and were engaging due to the format of having each participant evaluate images. Challenges of the hybrid course included blocking an hour during clinical service as most participants do not routinely have protected time. Hands-on teaching sessions were considered critical to developing competency with all participants requesting more frequent sessions during and following the course. Participants all reported lacking confidence in using POCUS in clinical care at course completion but are motivated to continue to develop this skill further.

## Discussion

The hybrid virtual and in-person I-ScaN POCUS course was as successful as a traditional course in improving learner knowledge, hands-on skill and confidence after approximately 8 months from beginning the course. Participants had a strongly favorable perception of the hybrid format.

Traditional POCUS courses were not allowed at our institution during the COVID-19 pandemic due to social distancing requirements for the classroom setting as well as with standardized patients. We decided to upload lectures recorded by our instructors rather than refer learners to textbooks or other online resources to foster a community atmosphere and build the culture of POCUS locally. The ability to rewatch these videos was perceived as a strength of the course and will continue to be offered even as in-person lectures are resuming.

Our hybrid instruction design builds on best practices for faculty development and innovative instructional design including use of spaced learning and flipped classroom approaches [13–15]. Online lectures could be accessed whenever and as often as people needed. Image interpretation sessions online were given at multiple times, accommodating participant schedules while avoiding the common barrier of needing instructors and participants to block days in a row for a traditional course that may also require travel. These results are relevant for POCUS education in LIMC and other settings, where barriers to traditional programs exist [8]

Studies of POCUS education for faculty and resident learners have demonstrated a similar need for a robust longitudinal curriculum following an introductory course [9, 16, 17]. The modular structure supported knowledge and skill retention through spaced learning, repetition of principles, and establishing positive scanning habits over time, features which were praised during focus group discussions.

A critical aspect of any POCUS course is to practice scanning on a person, either a patient or standardized patient [9, 18, 19]. Use of the simulation center was not allowed when the course began so we performed scanning sessions on hospitalized patients. Mitigation strategies to protect learners and patients were accomplished by holding the sessions during a period of low COVID-19 hospitalization rates when we could scan only COVID-19 negative patients in small groups wearing recommended PPE. We found that performing the hands-on scanning sessions with admitted patients instead of standardized patients created a richer learning environment given the pathology and body types encountered that are often lacking in the thin, male, healthy standardized patients most commonly employed in POCUS courses.

The two focus groups, one held with a cohort that completed the course and another with a cohort who was registered but did not participate, illuminate attitudes and perspectives about POCUS from a diverse and growing demographic of prospective POCUS adopters representing faculty in ambulatory settings, APPs and hospitalists. A striking finding was the lack of confidence in those who successfully completed the 8-month course, mirroring findings in the survey results from the traditional cohort. Learner confidence, though not significantly different between cohorts, remains a barrier to adoption of POCUS into clinical and educational practice. The early enthusiasm experienced by learners wanes with progression along the Dunning–Kruger curve as learners perceive the steep path required to achieve competency [20]. We have introduced additional POCUS workshops for learners to boost their knowledge, skill and confidence. These refresher workshops teach POCUS using a symptom-based approach using clinical case prompts that include abnormal images, allowing learners to practice views on healthy human models.

A recent study found that a 4-week tele-ultrasound course improved learner post-course knowledge as much as a traditional 2-day course [21]. Similar to our observation, the authors concluded the extended 4-week format led to improved knowledge retention, though were unable to perform hands-on skill assessments or determine retention of knowledge, skill or attitudes over time.

Study limitations include a relatively small and under-powered sample size of those completing the course. Participants in the traditional cohort were all hospitalists, while those in the hybrid cohort were from diverse backgrounds including APPs, surgical faculty and general medicine faculty. The small sample size precludes a deeper analysis of between-group differences. Repeating online sessions to accommodate participant schedules created a burden on the instructors and was logistically challenging. A typical session was held 4–6 times, including the make-up sessions. As we did not instruct participants to limit educational resources to those provided by our course we are unable to control for differences in ultrasound education the study subjects might have obtained.

## Conclusions

The success of the hybrid I-ScaN POCUS program elements of uploaded lectures, online quizzes, and online image interpretation sessions delivered in a modular format supports ongoing adaptation of these types of instructional design into future courses while continuing to emphasize hands-on supervised practice. The ability to incorporate an array of virtual options into POCUS courses to maximize safety, value and effectiveness will be of great benefit to course directors and learners alike.

## Supplementary Information

**Additional file 1:** NYU self-certification form.

## Data Availability

The data sets during and/or analyzed during the current study available from the corresponding author on reasonable request.

## Abbreviations

POCUS: Point-of-care ultrasound
APP: Advanced practice provider
SHM: Society of Hospital Medicine
NYU: New York University
I-ScaN: Integrated Sonography Course at NYU
PPE: Personal protective equipment
