# Supplementary material for: Design and comparison of a hybrid to a traditional in-person point-of-care ultrasound course
Source: Ultrasound J. 2022 Mar 12;14:12. doi: 10.1186/s13089-022-00261-x (PMC8917361; doi:10.1186/s13089-022-00261-x)
Supplement: Supplementary file 1 — Additional file 1: NYU self-certification form. [file 13089_2022_261_MOESM1_ESM.pdf]

Institutional Review Board
Human Research Regulatory Affairs
1 Park Avenue | 6th Floor | New York, NY 10016
http://irb.med.nyu.edu

Self-Certification Form

Determining Whether Your Proposed Activity is Quality Improvement (QI)

INSTRUCTIONS:

1. Use this form:
    a. if you think your proposed activity could be considered a quality improvement (QI) project and may not require IRB review
    b. if you are unsure whether or not you need to submit your project to the IRB

2. The conditions below must be met in order for your proposed activity to be considered QI that does not require IRB review. Do not submit this form to the NYU SoM IRB. This form may be used as documentation that your proposed activity is QI that does not require IRB review.

3. **THIS IS NOT AN IRB REVIEW**. If your proposed activity is considered human subjects research, you will need to submit it to the NYU SoM IRB for review via Research Navigator.

Information for the **Project Leader**			
Name and Degree(s)	Michael Janjigian, MD		
Phone Number	212-562-8360	E-mail Address	Michael.janjigian@nyumc.org
Institution and Department	NYU School of Medicine, Department of Medicine		
Mailing Address	462 First Ave, NB16N1, NY, NY 10016		
Project Title	NYU POCUS Hospitalist Training		

Determining if a project constitutes human subjects research or QI involves multiple factors. Although QI

activities may involve human participants, most are not considered research that requires IRB review. This is an important distinction to make because it determines whether IRB review and oversight of a project is needed. QI activities that are subject to IRB review and oversight are those that qualify as ***research*** and involve ***human subjects*** as defined by federal regulations.

RESEARCH is defined under 45 CFR 46.102(l) as a ***systematic investigation***, including research development, testing and evaluation, designed to develop or contribute to ***generalizable knowledge***. Activities that meet this definition constitute research for the purpose of this policy, whether or not they are conducted or supported under a program, that is considered research for other purposes.

Systematic investigation is an activity that involves a prospective research plan that incorporates data collection, either quantitative or qualitative, and data analysis to answer a research question. Systematic investigation will involve a predetermined method for studying a specific topic, answering a specific question(s), testing a specific hypothesis(es), or developing a theory.

Generalizable knowledge is designed to draw general conclusions, inform policy, or generalize findings. To develop or contribute to generalizable knowledge requires that the results (or conclusions) of the activity are intended to be extended beyond a single individual or an internal program.

HUMAN SUBJECT is defined under 45 CFR 46.102(e)(1) as a living individual about whom an investigator (whether professional or student) conducting research:

1) Obtains information or biospecimens through intervention or interaction with the individual, and uses, studies, or analyzes the information or biospecimens; or

OR

2) Obtains, uses, studies, or generates identifiable private information or identifiable biospecimens

Elements of QI Activity and Human Subjects Research: **Key Differences**		
**Points to Consider**	**QI Activity**	**Human Subjects Research**
Starting Point	To improve performance/care	To answer a question or test a hypothesis
Purpose	To assess process/program/system as judged by established/accepted standards	To develop or contribute to generalizable knowledge
Design	Adaptive	Follows a fixed protocol throughout the duration of the proposed work
Benefits	Directly benefits a process/program/system; may or may not benefit patients	May or may not benefit current subjects; intended to benefit future patients
Risks	No anticipated increase in risk to patients, with exception of possible privacy/confidentiality concerns	May put subjects at risk of harm
Participation Obligation	Responsibility to participate as component of care	No obligation for individuals to participate
Analysis	Compares a program/process/system to an established set of standards	To statistically prove or disprove a hypothesis
Adoption of Results	Promptly adopts results into local care delivery	Little urgency to disseminate results quickly
Publication	Clinicians are encouraged to share insights; results may be published	Investigators are obliged to share results

INSTRUCTIONS: Complete the following section to help you determine if your proposed activity falls in the realm of QI. If a statement is true, check off **YES**. If all of your responses to the below statements are positive (i.e., checked off **YES**), then your proposed activity constitutes QI that does not require IRB review or oversight.

	QI Certification Statements	YES	NO
1	Your activity's primary objective is to produce an improvement in safety or care that will be sustained over time at the local institution or within a particular program at the local institution.<br>***NOTE:*** *If the intended outcome is simply to report on what happened at the local institution/program, it does not indicate research design or intent as it may not be generalizable outside of the local institution.*	x☐	☐
2	Your activity does **NOT** use a fixed protocol for the duration of the proposed work.<br>***NOTE:*** *If frequent adjustments are needed, your answer should be "YES."*	x☐	☐
3	Your activity does **NOT** involve an intervention that may pose risks greater than those presented by routine clinical care.	x☐	☐
4	There will be minimal delays in implementing changes from results.	x☐	☐
5	All individuals involved in key project roles have on-going commitment to the improvement of the local care situation.	x☐	☐
6	Your activity is **NOT** funded by an outside organization with commercial interest in the use of the results.<br>***NOTE:*** *The purpose of this statement is to determine if the project has received funding to be conducted as a research study.*	x☐	☐
7	Your activity is **NOT** part of a multi-center project that involves non-NYUL Health sites.<br>***NOTE:*** *If it is being conducted in a multi-site context with a common protocol across sites, then the results may be generalizable and thus constitute research.*	x☐	☐

Certification for the Project Leader

Date	4/6/18
Print Name	Michael Janjigian
Signature	*I certify that the information provided above is accurate.*

NOTE: If the results of this form indicate your proposed activity is considered QI and not research involving human subjects, consistent with the federal regulations governing human subject research, IRB review or oversight is not required.
